# Mixed Metal Oxide W-TiO_2_ Nanopowder for Environmental Process: Synergy of Adsorption and Photocatalysis

**DOI:** 10.3390/nano14090765

**Published:** 2024-04-26

**Authors:** Khley Cheng, Socheata Heng, Siteng Tieng, Ford David, Sarah Dine, Oriana Haddad, Christophe Colbeau-Justin, Mamadou Traore, Andrei Kanaev

**Affiliations:** 1 Department of Chemistry, Royal University of Phnom Penh, Russian Blvd., Phnom Penh 120407, Cambodia; chengkhley@yahoo.com (K.C.); socheataheng3579@gmail.com (S.H.); tiengsiteng@gmail.com (S.T.); forddavidaus@gmail.com (F.D.); 2 Laboratoire des Sciences des Procédés et des Matériaux, CNRS, Université Sorbonne Paris Nord, 93430 Villetaneuse, France; sarah.dine@lspm.cnrs.fr (S.D.); oriana.haddad@cnrs.fr (O.H.); mamadou.traore@lspm.cnrs.fr (M.T.); 3 Institut de Chimie Physique, CNRS UMR 8000, Université Paris-Saclay, 91405 Orsay, France; christophe.colbeau-justin@universite-paris-saclay.fr

**Keywords:** W-TiO_2_ nanopowder, adsorption, photocatalysis, UV-A, sunlight, synergy between processes

## Abstract

A mixed metal oxide W-TiO_2_ nanopowder photocatalyst was prepared by using the sol–gel method with a broad range of elemental compositions x = C_W_/(C_W_ + C_Ti_), including TiO_2_ and WO_3_. The material was structurally characterized and evaluated in adsorption and photocatalytic processes by testing its removal capacity of a representative pollutant methylene blue (MB) in aqueous solutions and under UV-A and sunlight illuminations. The nanopowders appeared to be more effective adsorbents than pure TiO_2_ and WO_3_ materials, showing a maximum at 15 mol% W, which was set as the tungsten solubility limit in anatase titania. At the same time, the photocatalytic decomposition of MB peaked at 2 mol% W. The examination of different compositions showed that the most effective MB removal took place at 15 mol% W, which was attributed to the combined action of adsorption and heterogeneous photocatalysis. Moreover, MB decomposition under sunlight was stronger than under UV-A, suggesting photocatalyst activation by visible light. The pollutant removal efficiency of the material with 15 mol% W was enhanced by a factor of ~10 compared to pure TiO_2_ at the beginning of the process, which shows its high potential for use in depollution processes in emergency cases of a great pollutant leak. As a result, a W_x=0.15_-TiO_2_ catalyst could be of high interest for wastewater purification in industrial plants.

## 1. Introduction

The research on TiO_2_ photocatalysts is steadily increasing since the discovery of UV-light assisted electrochemical water splitting [1]. The extensive research has shed light on the relevant mechanisms and reaction pathways [2,3,4]. As a result, more than four million tons of TiO_2_ per year are nowadays produced to satisfy the growing research and industrial demand in environmental and energy-related fields [5,6].

A TiO_2_ photocatalyst is an attractive material because of its physical and chemical stability, safety, resistance to photocorrosion, low cost, non-toxicity, large specific surface area and oxidation strength [7,8,9,10,11,12,13,14,15,16,17,18,19]. However, its insignificant activity under visible light requires improvements. Furthermore, the limiting adsorption of TiO_2_ sets restrictions on its use in emergency cases of a great pollutant leak. There have been many attempts made to improve the photocatalytic activity of TiO_2_ by changing the size of the particles, modifying the band gap energy, postponing the recombination of electrons and holes, combining it with adsorbents (e.g., activated carbon [20]), etc. As a method to improve its activity, the mixing of TiO_2_ with other metal oxides, such as CuO, V_2_O_5_, ZrO_2_, Co_3_O_4_, Fe_2_O_3_, ZnO, MnO_2_ and WO_3_, has been undertaken [21,22,23,24,25,26,27,28,29,30]. These inorganic materials are reuseable and highly sustainable. Among them, transition metal oxide tungsten trioxide WO_3_ is one of the potential attractive materials because of its relatively narrow band gap (2.4–2.8 eV) and deep valence band (+3.1 eV) energies, but also due to its low cost, photocorrosion stability in aqueous solutions, non-toxicity, electrical conductivity, and reversibility of transformation between W(VI) and W(V) [31,32]. It has been studied in different applications such as photocatalysis, electrochromic and field emission devices, smart windows, thermal control of satellites and gas sensors [33,34]. The admixing of WO_3_ is considered effective in improving the photocatalytic activity of TiO_2_, since W(VI) conversion to W(V) functioning as an electron acceptor increases the efficiency of photoinduced charge (e^−^/h^+^) separation [35]. The spatial separation of photoinduced charges reduces their recombination rate, thus enhancing photocatalytic activity [36]. Furthermore, according to Lewis and Bronsted, acidic W^6+^ readily adsorbs OH^−^ and/or H_2_O and is considered effective in producing OH^•^ radicals that destroy organic contaminants [28]. Because of the lower band gap energy (E_g_ = 2.4–2.8 eV) compared to anatase TiO_2_ (3.2 eV), WO_3_ absorbs visible light [37,38] and can extend the activity spectral range of the sunlight. The mixed metals oxide W-TiO_2_ has already been considered for anti-fogging surfaces, self-cleaning glasses, self-cleaning superhydrophylic surfaces [39], photocatalysis [21,28,29,35,36,38,40,41,42,43,44,45,46,47,48,49,50,51,52,53,54], charge storage [55], solar energy conversion [44], ethylene sensors [56], air treatment [57], anti-corrosion purposes [58], hydrogen production [19,59,60], smart glass industry [61], wastewater treatment [62], energy storage [63,64,65], humidity sensors [66] and photo-electrochemistry [67]. The applications of W-TiO_2_ photocatalysts for pollutant removal in aqueous solutions are summarized in Table 1.

W-TiO_2_ composites have been prepared via several methods including physical mixing [68], sol–gel [7,19,21,29,35,36,37,38,48,69,70,71,72,73,74], solution combustion [50], hydrothermal [52,55,75,76,77], ultrasound-assisted [28], electrospinning [47,78], impregnation [42], wet chemical [63] and incipient wetting [21,51]. Among these methods, the sol–gel technique is proven to be highly attractive for synthesis because of its uncomplicated, low-cost procedure and flexible synthetic routes and the possibility of attaining highly homogeneous compositions [37,79]. At the same time, the variation in adsorption in the mixed metal oxide remains underexplored. An available study [80] has applied an addition of W to TiO_2_ Degussa P25 powder, which did not allow for the formation of perfectly homogeneous compounds at the nanoscale. A competition between an increase in the specific surface area and a decrease in the activity per adsorption site has been also confirmed in the compounds with W content ≤ 6 mol% [81]. However, the elemental composition can greatly affect the specific surface area of these materials, as it has been shown in an example of Zr_x_Ti_1−x_O_2_ nanopowders [23]. This offers an opportunity to control adsorption and photocatalysis processes in the same material. The full range of elemental W/Ti compositions has not been explored in previous studies with respect to the adsorption and heterogeneous reactions’ effectiveness, covering UV-A (most suitable for pure titania) and sunlight illuminations for the photocatalyst activation. 

In this work, we report on a simplified preparation procedure of mixed metal oxide W-TiO_2_ nanopowders via the sol–gel method with a broad range of elemental compositions. The adsorption and photocatalytic activity of the prepared materials were tested with UV-A lamp and natural sunlight illuminations based on the removal of a methylene blue (MB) pollutant in aqueous solutions. The influence of the material composition on the process effectiveness was inspected.

## 2. Materials and Methods

### 2.1. Synthesis Procedure

All chemicals used in this study were analytical grade. Titanium isopropoxide (TTIP, TiO_4_C_12_H_32_, >98% purity) and tungsten (VI) chloride (WCl_6_, >99.9% purity) were bought from Acros Organics (Geel, Belgium). 2-propanol of HPLC grade (≥99.9% purity) and MB were purchased from Sigma-Aldrich (manufactured by Merck KGaA, Darmstadt, Germany). 

**Figure 1:**
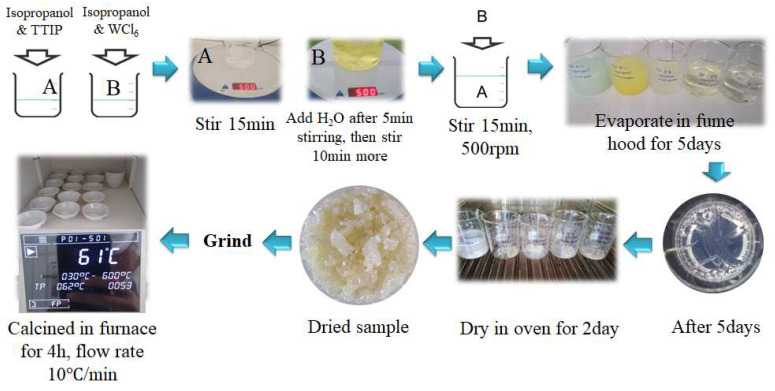
Procedure for synthesis of W-TiO_2_ nanopowders. A and B are isopropanol solutions of TTIP and WCl_6_, respectively.

W-TiO_2_ nanopowders were synthesized via the sol–gel method. A schematic diagram of the W-TiO_2_ nanopowder preparation procedure is depicted in Figure 1. The synthesis was conducted at the total precursor concentration of C_Ti_ + C_W_ = 0.3 mol/L with a hydrolysis ratio h = C_H2O_/(C_Ti_ + C_W_) = 1.25, where C_H2O_, C_Ti_ and C_W_ are molar concentrations of water, TTIP and tungsten (VI) chloride. The pure TiO_2_ powder was prepared by dissolving TTIP in 2-propanol under stirring at 500 rpm for 15 min to obtain solution A. Then, the required amount of water was mixed with 2-propanol under stirring for 15 min to obtain solution B, which was slowly dropped into solution A under intense stirring, which triggered the nucleation of titanium oxo-alkoxy (TOA) nanoparticles [82]. The obtained colloidal solution was dried in a fume hood at room temperature for 5 days to obtain white amorphous powders, which were further dried in an oven at 85 °C for 2 days and afterwards calcined in a Nabertherm furnace (Lilienthal, Germany) at 450, 500, 550 and 600 °C for 4 h. The pure WO_3_ powder was prepared by dissolving an appropriate amount of WCl_6_ in 2-propanol under stirring for 15 min to obtain solution A. Solution B was prepared by adding the required volume of water to 2-propanol and then stirring for 15 min. After that, solution B was slowly dropped into solution A under stirring for 15 min. The mixed solution was dried, evaporated and calcined in the same way as the pure TiO_2_. The mixed metal oxide W-TiO_2_ nanopowders were prepared with different compositions x = C_W_/(C_W_ + C_Ti_) = 0.0025, 0.01, 0.02, 0.04, 0.06, 0.08, 0.15, 0.30 and 0.50. Solution A was prepared by following the preparation procedure of pure TiO_2_. For solution B, the required amount of WCl_6_ was dissolved in 2-propanol under stirring for 5 min, and then water was added under stirring for 10 min. Solution B was then slowly dropped into solution A under stirring, then dried, evaporated and calcined in a furnace at 450, 500, 550 and 600 °C for four hours. Equal volumes of the solutions A and B were used in all synthesis procedures. The total volume of 150 mL was used for compositions x = 0, 0.0025, 0.01, 0.02, 0.04, 0.06 and 0.08, 100 mL for compositions x = 0.15 and 0.30, 50 mL for x = 0.50, and 20 mL for x = 1. 

### 2.2. Characterization Techniques

The X-ray diffraction (XRD) patterns were collected with a Co-Kα, INEL EQUINOX 1000 apparatus. The specific surface area of samples was measured by N_2_ physisorption at 77 K using a BELSORP-MAX apparatus from Microtrac (Haan, Germany). The specific surface area was obtained over the relative pressure range (0.05–0.25) using the Brunauer, Emmet and Teller (BET) equation. Before analysis, samples were degassed under primary vacuum (<150 mPa) at 393 K for 12 h using a BELREP VAC module from Microtrac. The Raman spectra were measured with a high-resolution micro-Raman spectrometer HR800 HORIBA Jobin Yvon (Palaiseau, France) operating at a wavelength of 473 nm, with spectral and spatial resolutions, respectively, of 0.25 cm^−1^ and 5 μm. The scattered light was collected in a backscattering configuration and recorded on a Peltier-cooled CCD camera. EDX measurements were performed with a Hitachi TM3000 TableTop Scanning Electron Microscope (Tokyo, Japan) equipped with a Swift ED3000 module for elemental analysis. 

The time-resolved microwave conductivity (TRMC) measurements were conducted at the experimental installation described in Ref. [83]. The method allows for the monitoring relaxation of the photoinduced charges by monitoring changes in the microwave power reflected by a semiconductor surface after light illumination, which induces electrical conductivity. In these experiments, the samples were irradiated with 360 nm light pulses of 8 ns duration and 1.5 mJ energy delivered by a cw OPO Nd:Yag laser (EKSPLA) operating at a 10 Hz repetition rate. The area of samples with a 5 mm diameter was illuminated and the recorded TRMC decay curves were accumulated over 200 laser pulses. The measurements were performed at room temperature and in an ambient atmosphere.

The adsorption capacity of 0.025 g of W-TiO_2_ nanoparticles was measured in aqueous solutions of 100 mL volume with 10 ppm MB. The solution was stirred at 500 rpm in the dark and then 5 mL of the solution was taken every 15 min into a centrifuge tube. This sample was centrifuged at 4300 rpm for 15 min using the CompactStar CS4 setup to precipitate the powders, and the concentration of the remaining MB in the clear solution was calculated after measurements of light absorption at λ = 664 nm using a UV–visible spectrophotometer HS 3300 (Humas, Daejeon, Korea). 

The photocatalytic activity was measured through the decomposition of 10 ppm MB in aqueous solutions of 200 mL volume, with 0.025 g of W-TiO_2_ nanoparticles. The catalyst powder was added to the MB solution under stirring at 500 rpm in the dark until adsorption equilibrium was reached and the solution was then illuminated with a UV-A lamp VL-6.LC (λ = 365 nm, 24 W; VWR International, Rosny-sous-Bois, France) at a distance of 10 cm. The photocatalytic activity under sunlight illumination was tested between 9 and 12 am in Phnom Penh city (Cambodia) with the same nanoparticle concentration of 0.125 g/L. In these experiments, the full series of W-TiO_2_ photocatalysts with different elemental compositions (or W content, equal to 100× in mol%) was placed in an identic aqueous solution of MB pollutant and simultaneously exposed to sunlight or a UV-A lamp. Samples of 5 mL of the solution were taken every 15 min and centrifuged at 4300 rpm for 15 min in order to separate catalyst particles, and absorption at λ = 664 nm was measured by the UV/visible spectrophotometer to evaluate the remaining MB concentration. The first-order rate constants of MB decomposition were measured for a comparison of the material activities.

## 3. Results and Discussion

### 3.1. Structural Properties

Before discussing the functional properties of the prepared materials, some comments will be given on their structural properties. We notice that nanopowders prepared according to the described method are expected to consist of the smallest nanoparticles—nuclei. Indeed, this fact has been previously evidenced in TOA nanoparticles formed at low hydrolysis ratios h ≤ 1.5 [80,81]. These TOA nuclei have the size of 2R = 3.2 nm and agglomerate into larger particles while keeping their individual characteristics [84]. The nucleation growth process of tungsten–titanium oxo-alkoxy (WTOA) nanoparticles in the sol–gel synthesis procedure has not been yet documented. However, the WCl_6_ precursor is known to readily hydrolyze. Keeping in mind the stronger Pauling electronegativity of W (2.36) compared to Ti (1.54), one can expect a faster hydrolysis rate compared to the polycondensation of tungsten species, similar to vanadium species. The last point argues for a similar tendency in the formation of composite WTOA nanoparticles to that of vanadium–titanium oxo-alkoxy (VTOA) nanoparticles [85]. Although perfect micromixing conditions were not controlled in the simplified synthesis procedure, an extremely low hydrolysis ratio well below that required for the complete nucleation slowed down the reaction kinetics, making quasi-perfect elemental dispersion possible at the nanoscale, probably forming single-shell and core–shell WTOA nanoparticles depending on the colloid composition. Future studies will clarify this issue. We also confirmed the elemental compositions x of W-TiO_2_ nanopowders after the synthesis procedure by performing EDX measurements (Table S1 and Figure S1 of Supplementary Materials).

**Figure 2:**
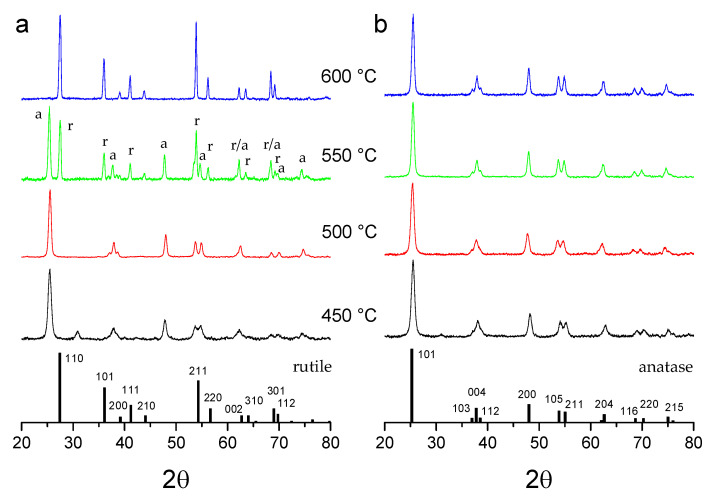
X-ray diffraction patterns of TiO_2_ nanopowders with 0 mol% (**a**) and 1 mol% (**b**) W doping after calcination at temperatures between 450 and 600 °C. Vertical bars show positions and heights of anatase (a) and rutile (r) peaks.

XRD patterns of the calcined W-TiO_2_ nanopowders showed evidence of a general tendency to stabilize the metastable anatase phase. The most intense peaks of the anatase (2θ = 25.28°) and rutile (2θ = 27.44°) phases can serve as a guide to follow the phase transformation. As Figure 2a shows, the stable rutile phase appeared in pure TiO_2_ after calcination at temperatures above 500 °C, which is in agreement with previous studies [86]. 

In contrast, no traces of rutile were observed in W-doped nanopowders even at 600 °C (Figure 2b). Besides the stabilizing interaction, this indicated a very homogeneous elemental mixing of W and Ti elements at the nanoscale, which led to no pure titania domains transforming to the rutile phase. 

**Figure 3:**
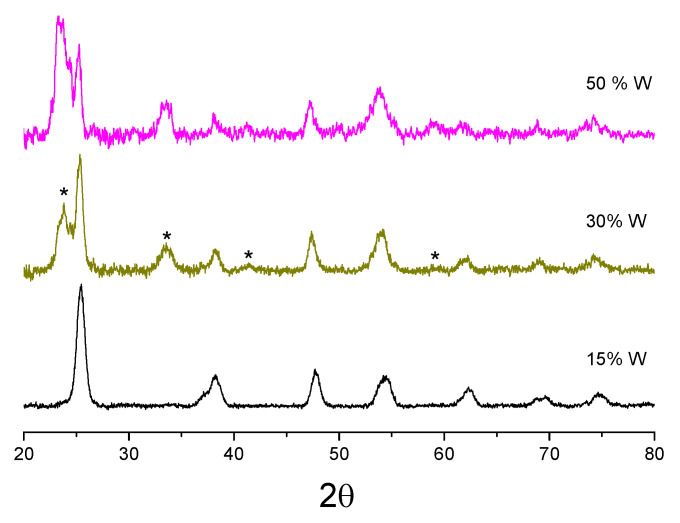
X-ray diffraction patterns of TiO_2_ nanopowders with 15, 30 and 50 mol% W after calcination at 550 °C. Main peaks belong to anatase TiO_2_; appearing monoclinic WO_3_ is marked by (*).

Furthermore, the anatase TiO_2_ crystalline phase was preserved with W insertion up to 15 mol%, as evidenced Figure 3. In contrast, with a greater W loading, WO_3_ crystallized in the monoclinic phase [87], as shown in the growing peaks at 23.5° and 33.5°. This behavior was common to all prepared powders, and the fraction of the WO_3_ phase increased with an increase in the temperature from 500 °C to 600 °C. It has been earlier shown that additives (Li^+^, K^+^, Cu^2+^ and Al^3+^) inducing vacancy in the anion sublattice promote the anatase–rutile phase transition, while additives (S^5+^, P^5+^ and Nb^5+^) reducing the number of vacancies inhibit the phase transition [88]. This effect has also been observed in 1–4 mol% W-doped TiO_2_ [89]. Our experimental findings support these observations in a larger domain of the material compositions.

**Figure 4:**
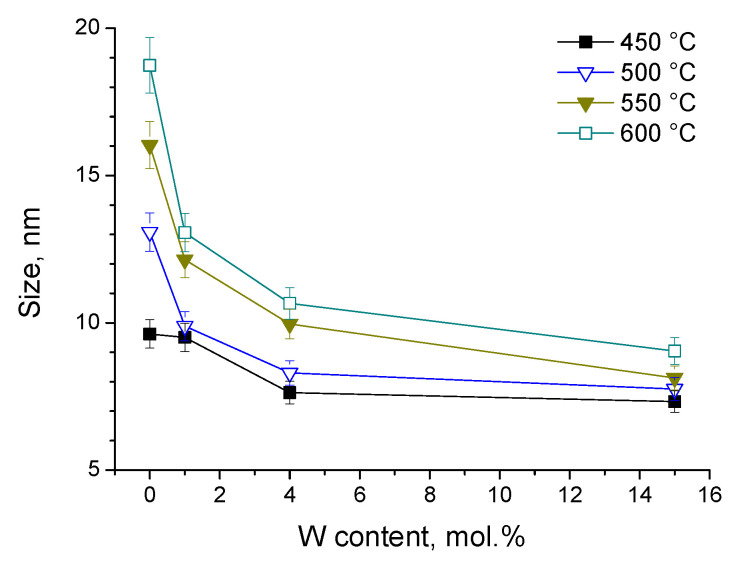
Crystalline size of W-TiO_2_ powders versus W content after calcination at different temperatures (obtained from XRD patterns by Scherrer equation).

Changes in the nanopowder morphology accompanied the particles’ crystallization. In particular, a clear decreasing trend in crystallite size was observed with an increase in W content, as Figure 4 shows based on the Scherrer equation. The decrease in the crystallite size of Ti_0.8_W_0.2_O_2_ compared to pure titania has been previously reported and attributed to the incorporation of W into the TiO_2_ lattice [90].

**Figure 5:**
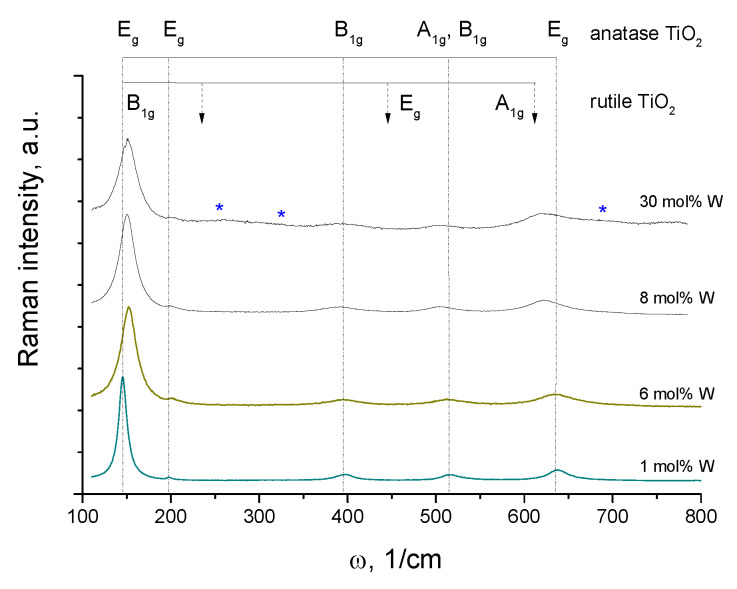
Raman spectra of W-TiO_2_ nanopowders with different W contents after calcination at 550 °C. Positions of appearing WO_3_ bands at 30 mol% W are shown by (*).

Two particularities of the prepared materials can be observed from the Raman analysis in Figure 5. The first one is that the low-energy E_g_ mode in the range of ~150 cm^−1^ shifts to higher frequencies upon W insertion, which has been previously attributed to the unit cell expansion [91]. However, (in disagreement with this last study) no linear shift in this Raman mode with the increase in the W content, saturating at 15 mol% W, was observed in our work. In contrast, the shift appeared to be significant between 1 and 6 mol% W, with no subsequent evolution upon greater W insertion. The second particularity concerns the blue shift in the low-frequency A_1g_/B_1g_ and high-energy E_g_ modes with an increase in W content above 6 mol%. This may be an indication of the cell deformation toward rutile geometry. In agreement with the XRD analysis, the Raman spectra (broad bands at 260, 320 and 690 cm^−1^, marked by (*) in Figure 5) confirm the material segregation above 15 mol% W, resulting in anatase TiO_2_/monoclinic WO_3_ [92] polymorph powders. In contrast, the dissolution of W into te anatase TiO_2_ matrix is complete for the lower W contents. Because of that, we tentatively ascribed 15 mol% to the solubility limit of tungsten in anatase titania. 

### 3.2. Adsorption Capacity

**Figure 6:**
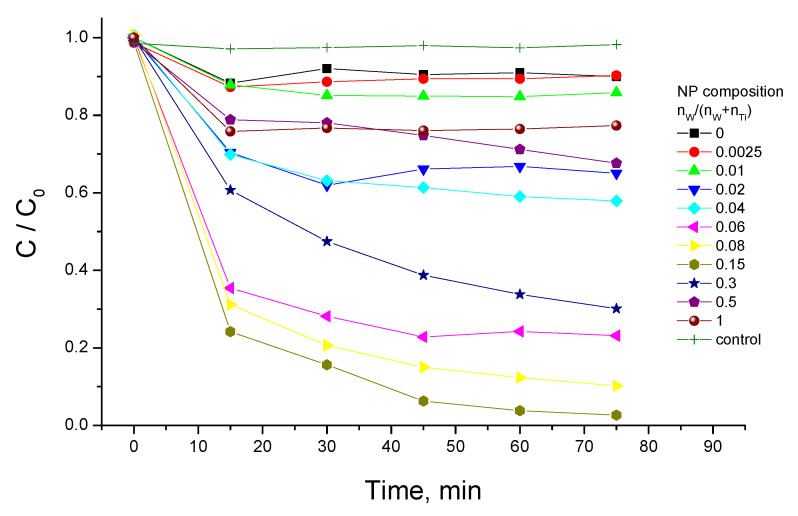
MB adsorption kinetics of W-TiO_2_ nanopowders calcined at 550 °C (*C_catalyst_* = 0.25 g/L).

The MB adsorption by W-TiO_2_ nanopowders of different compositions calcined at 550 °C is shown in Figure 6 in terms of C/C_0_, where C and C_0_ are, respectively, the remaining and initial MB concentrations in the solution. It can be seen that W-TiO_2_ materials possess a stronger MB adsorption capacity than pure TiO_2_. The strongest adsorption was observed in the material with 15 mol% W, which was about 50 times above that of pure TiO_2_. This adsorption enhancement is significantly higher than that previously reported in W-TiO_2_ powders prepared through ammonium metatungstate liquid deposition onto TiO_2_ Degussa P25 powder, where an eightfold increase in MB adsorption has been reported in a 6.5 mol% W material [80]. This difference in both optimal composition and enhancement factor can be clearly attributed to the more homogeneous W distribution in the material in our synthesis conditions, where W species interact with much smaller reactive TOA nuclei of 3.2 nm size [84] compared to tens of nanometers of TiO_2_ Degussa P25 [93]. The highest adsorption capacity of W-TiO_2_ nanopowder was 32 mg/g of MB, attained after 15 min of the experiment. This corresponds to the adsorption capacity of the coal-based activated carbon in treating high-salt printing and dyeing wastewater [94], which, however, showed significantly slower kinetics. This shows good potential of the prepared materials in environmental processes.

**Figure 7:**
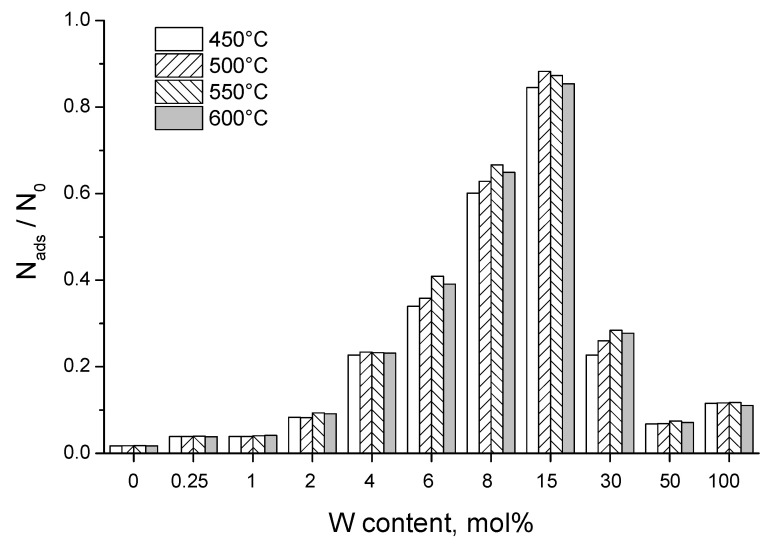
Equilibrium absorbance (Lagergren model) of W-TiO_2_ nanopowders with different compositions calcined at 450, 500, 550 and 600 °C (*C_catalyst_* = 0.125 g/L).

The adsorption abilities of W-TiO_2_ nanopowders calcined at different temperatures of 450, 500, 550 and 600 °C were further compared. The equilibrium absorbance N_ads_/N_0_ according to the Lagergren model [95] and Langmuir isotherm correction of the photocatalysts’ mass (equal to 0.125 g/L, as that used in the photocatalytic MB decomposition experiments) is plotted in Figure 7. As one can see, the adsorption capacity of the prepared W-TiO_2_ nanopowders increased with an increase in W content up to 15 mol% and then reduced with the greater W loadings. The maximum adsorption capacity of 0.25 mM/g is 2.5 times larger compared to that previously reported [80]. At the same time, we noticed that the adsorption of the prepared materials was not significantly affected by the calcination temperature.

We noticed that the specific surface area (σ) of the prepared materials measured by using the BET method (Figure S2 of Supplementary Materials) confirmed the tendency in their MB adsorption capacity: σ = 50 ± 3 m²/g in nanopowders with 0 ≤ C_W_/(C_W_ + C_Ti_) ≤ 4 mol% calcined at 550 °C, while it sharply increased to 171 m²/g in nanopowders with 15 mol% W. Following the decreasing tendency in crystallite size (Figure 4), these measurements showed the important modifications in the porosity of the material with the composition W_x=0.15_-TiO_2_, which has already been reported in mixed oxide materials [96]. Otherwise, the peak of the porosity correlates with the presumed solubility limit of W in the TiO_2_ matrix (see the XRD measurements) and may be connected to the phase separation phenomena at the component segregation point. This issue needs further investigation.

### 3.3. Photocatalytic Activity

**Figure 8:**
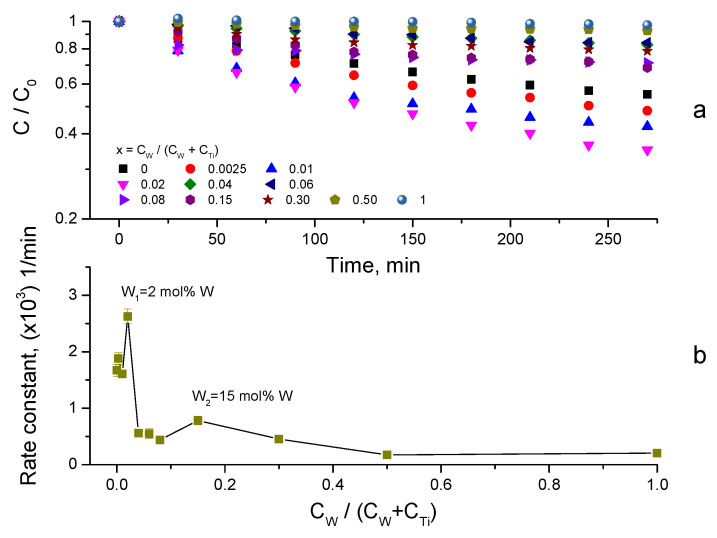
MB degradation kinetics using W-TiO_2_ photocatalyst under UV-A lamp (λ = 365 nm) illumination (**a**), and decomposition rate dependence on W content (**b**) (calcination temperature of 550 °C; *C_catalyst_* = 0.125 g/L).

After completing the adsorption measurements, the photocatalytic activity of the W-TiO_2_ nanopowders was investigated. These measurements were performed with the light source “on” after attaining the equilibrium of adsorption kinetics (Section 3.2). The results of MB removal using photocatalysts annealed at 550 °C with UV-A lamp illumination are shown in Figure 8a. The MB degradation kinetics followed first-order reaction kinetics ln(C/C_0_) = −kt, where C_0_, C, k and t are, respectively, the initial and remaining pollutant concentrations, the rate constant and the process time (see Figure S3 of Supplementary Materials). The obtained rate constants were used as a measure of the photocatalysts’ activities, which are shown in Figure 8b versus the composition x of the synthetized compounds. The materials’ activity exhibits two maxima, at low and high tungsten contents. While the strongest one observed at 2 mol% W has already been reported [80,91], the second maximum at 15 mol% W is new. The low doping of TiO_2_ by different cations generally produced an activity increase, in which the contribution of the photoinduced charge separation seems to play an important role [24]. On the other hand, the compositions corresponding to maximum adsorption (Figure 7) have not been reported as highly active so far. We also notice negligible direct MB photolysis by UV-A light.

**Figure 9:**
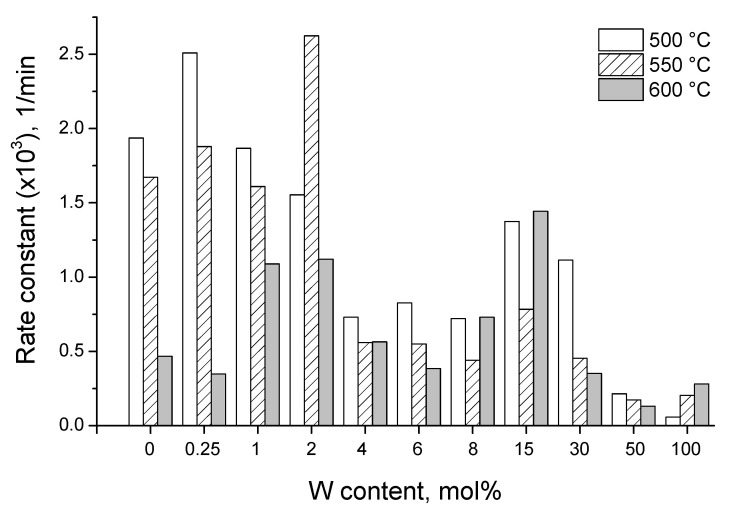
Photocatalytic activity of UV-A illumination (λ = 365 nm) on W-TiO_2_ photocatalysts with different compositions calcined at 450, 500, 550 and 600 °C (*C_catalyst_* = 0.125 g/L).

The photocatalytic rate constants of the prepared W-TiO_2_ nanopowders calcined at different temperatures are shown in Figure 9. One can see that the nanopowder activity does not correlate with that of the most active TiO_2_ component. Indeed, the pure TiO_2_ catalyst had the highest activity after calcination at temperatures of 450–500 °C, which corresponds to the pure anatase phase formation; in contrast, its calcination at elevated temperatures of 550 and, most evidently, at 600 °C resulted in an activity decrease, which is explained by the rutile phase formation [97]. Furthermore, an addition of tungsten extends the optimal activity to 550 °C (2 mol% W). Except for the activity increase upon low cation doping of a few mol% (followed by the activity decrease) taking place in many cation-doped TiO_2_-based materials, an evident second greatest enhancement in activity appearing around the concentration of 15 mol% clearly showed the added value of the mixed-oxide material.

**Figure 10:**
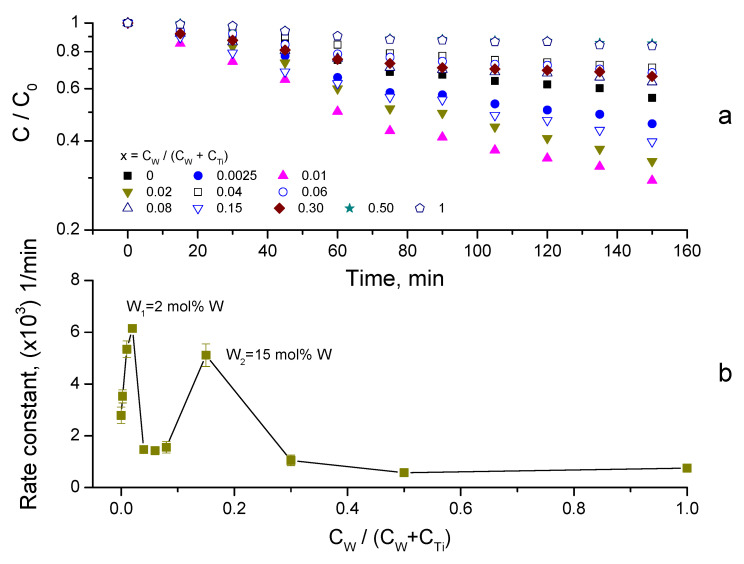
MB degradation kinetics using W-TiO_2_ photocatalyst powders (calcination temperature of 550 °C; *C_catalyst_* = 0.125 g/L) under sunlight illumination (**a**), and decomposition rate dependence on tungsten content (**b**).

The photocatalytic activity of the prepared W-TiO_2_ materials was additionally evaluated with natural sunlight illumination. In order to soften the influence of the daily sunlight intensity variations, these experiments were carried out simultaneously at the same time for the complete series of W-TiO_2_ nanopowders with different compositions. The results of the MB decomposition kinetics are shown in Figure 10a for the material calcined at 550 °C. The data followed first-order kinetics (Figure S3 of Supplementary Materials), and the rate constants are presented in Figure 10b as a function of the composition x of the synthetized compounds. 

The comparison of the W-TiO_2_ photocatalysts’ activities under sunlight (Figure 10b) and UV-A (Figure 8b) illuminations confirms the existence of two maxima, with a tungsten content of W_1_ = 2 mol% and W_2_ = 15 mol%. At the same time, the relative peak values of the corresponding rate constants are dependent on the light source. Indeed, while with UV-A illumination the rate constant k_UV_(W_1_) = 2.6 × 10^−3^ min^−1^ was significantly higher than k_UV_(W_2_) = 7.8 × 10^−4^ min^−1^, the materials’ activities with sunlight illumination were both enhanced and close to each other: k_SL_(W_1_) = 6.1 × 10^−3^ min^−1^ versus k_SL_(W_2_) = 5.1 × 10^−3^ min^−1^. Because, as previously discussed, W_2_ composition corresponds to the peak adsorption, the peak in the photocatalytic activity might be related to the direct photolysis of the adsorbed MB molecules (~97% according to Figure 6) by sunlight. However, we reject this possibility because of the negligible MB decomposition in the blank photocatalytic tests. As a result, we attribute k_SL_(W_2_) to the heterogeneous photocatalytic decomposition of MB by W_x=0.15_-TiO_2_ nanopowders.

### 3.4. Synergy of Adsorption and Photocatalysis

The heterogeneous photocatalytic process basically includes two steps of (1) physical adsorption of pollutant molecules on the material surface and (2) surface reactions including the release of reaction products. Since, in common experiments, the material activation by light is applied after attaining adsorption equilibrium, the contributions of these steps can be, respectively, characterized by the adsorbed amount of material C (in ppm units) and the apparent reaction rate constant k (in min^−1^ units).

**Figure 11:**
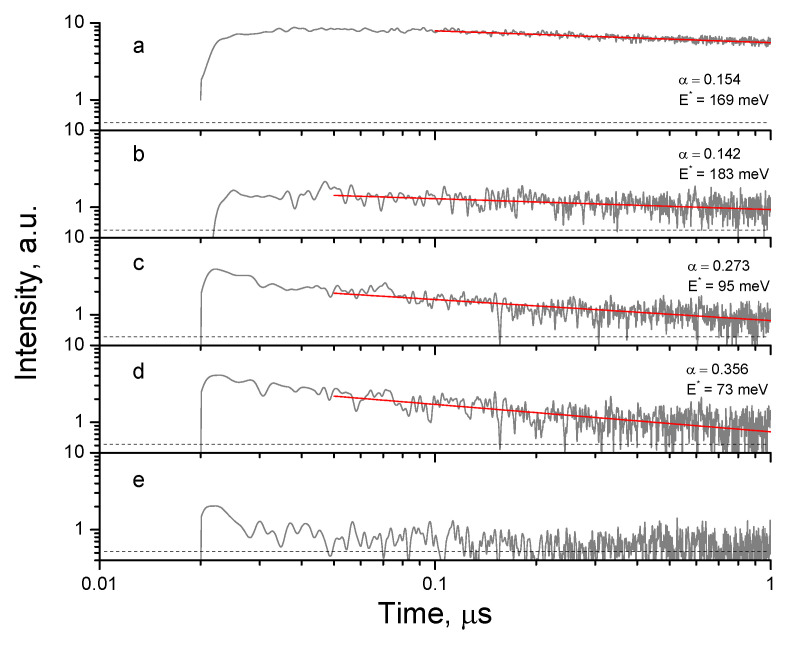
Photoinduced electron decay in pure TiO_2_ (**a**) and Ti_1-x_W_x_O_2_ powders with W content x = 1 mol% (**b**), 4 mol% (**c**), 15 mol% (**d**) and 100 mol% (**e**) calcined at 550 °C. Solid lines show least-squared fits with power law: I = At^α^ (power constants α and calculated electron trap energies E^*^ are indicated).

In order to inspect the reaction constant of the materials with different compositions, TRMC measurements were performed. The decay curves of the photoinduced charges in pure and W-doped TiO_2_ nanopowders are shown in Figure 11. The observed signal has been previously attributed to the liberation of charges from shallow traps with binding energy E^*^ following a power law decay I = At^α^ with parameter α = k_B_T/E^*^, where k_B_ is the Boltzmann constant and T is temperature [23]. The shallow states have attracted much attention over the last decade because of their key influence on the photocatalysts’ activity [98,99]. According to our data, the depths of the shallow electron traps increased from 169 meV (0 mol% W) to 183 meV (1 mol% W), while they decreased afterwards to 95 meV (4 mol% W) and 73 meV (15 mol% W). In nanopowders with x = 100 mol% (W-only material), the signal disappeared very rapidly to the noise level. Although no quantitative relation between the charges’ lifetime and photocatalytic rate has been proposed in previous studies, the present TRMC measurements confirm the observed tendency in the activity of the prepared powders, showing that photoinduced charges do not effectively trigger the reactions when they disappear fast.

A comparison with previous results summarized in Table 1 showed a general agreement about the enhancement factor of 2.5 ± 0.5 after the insertion of ~2 mol% W into TiO_2_, with both UV-A and sunlight illuminations. According to the TRMC measurements, the intrinsic material reactivity can be responsible for the maximum activity W_1_ (Figure 8 and Figure 10) but cannot be the only reason explaining the W_2_ maximum activity. Below, we discuss this issue in more detail.

**Figure 12:**
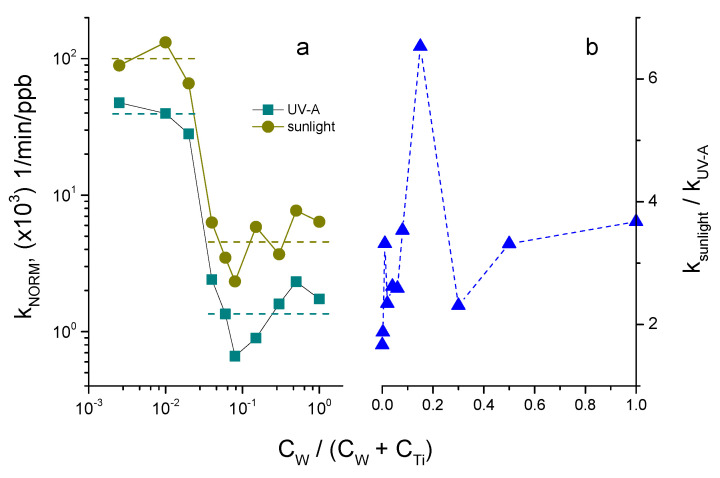
Normalized decomposition rate constant k_NORM_ for adsorbed MB by W-TiO_2_ photocatalyst calcined at 550 °C under UV-A and sunlight illuminations (**a**), and ratio of normalized rate constants under sunlight and UV-A illuminations (**b**).

The material activity in Figure 8 and Figure 10 generally reflects the combined action of photocatalytic and adsorption processes. In order to distinguish these two contributions, we normalized the decomposition rate constants (in min^−1^ units) for the adsorbed component (in ppb units) and plotted k_NORM_ (in min^−1^ppb^−1^ units) versus the tungsten content in the W-TiO_2_ nanopowders in Figure 12a. This rate constant k_NORM_ apparently reflects a pure photocatalytic response of the material to the adsorbed MB pollutant. Two particularities can be noticed:

– k_NORM_ was greatly different for 0 ≤ C_W_/(C_W_ + C_Ti_) ≤ 0.02 and 0.04 ≤ C_W_/(C_W_ + C_Ti_) ≤ 1;
– k_NORM_ was stronger under sunlight illumination compared to UV-A, attaining the peak value at 15 mol% W.

As we can see, the insertion of W into the TiO_2_ matrix does not enhance the intrinsic response of the mixed oxide material and the photocatalytic activity is most effective on pure anatase TiO_2_. Furthermore, the material with a W content of 4 mol% and higher has a greatly reduced k_NORM_, which drops down by about 30 times to a lower level. 

At the same time, the pollutant decomposition takes place at the material surface and the decomposition kinetics can be enhanced by increasing the number of pollutant molecules at the photocatalyst surface. A strong increase in the material adsorption due to the compositional adjustment may therefore promote synergy between the two processes (adsorption and reaction), moving toward the highest total effectiveness of the pollutant removal process. Taking into account a much stronger (by almost 100 times) initial elimination rate of MB via adsorption (Figure 6) compared to photocatalysis (Figure 8 and Figure 10), one can suggest preferential use of 15 mol% W-doped TiO_2_ nanopowders in the environmental process, especially in emergency cases. Indeed, a large amount of a pollutant can be rapidly bound to the photocatalyst surface in the first few minutes after an accident, while its decomposition can be readily achieved within a longer timescale. This would significantly reduce the negative impact of an accident on nature. 

The reaction inhibition in W-TiO_2_ nanopowders (see in Figure 12a), which probably is due to faster recombination of photoinduced electron–hole pairs (as confirmed by the TRMC data in Figure 11), can be largely compensated for by the material’s adsorption rate and capacity. The synergy factor (R) earlier proposed for the two-component composite TiO_2_-AC (AC = activated carbon) materials [20] cannot be directly applied to the one-component system, in which both reaction and absorption rates are tuned via elemental composition. However, taking it as a ratio of the pollutant removal rates R = k(W-TiO_2_)/k(TiO_2_), one can obtain R~10 at the beginning of the process, converging to R~2 after longer durations of the stationary operation under illumination, when adsorption equilibrium is attained. This shows the high potential of this material for depollution use in emergency cases of an occasional pollutant leak. The very high concentration of adsorbed pollutant molecules can largely compensate for the reduced reaction constant and results in effective pollutant removal. As a result, the W_x=0.15_-TiO_2_ nanocatalyst could be highly promising for wastewater purification in industrial plants.

The observed difference in the photocatalytic effectiveness of the W-TiO_2_ materials under UV-A and sunlight illuminations (Figure 12b) seems to be a general feature (see, e.g., V-TiO_2_ photocatalyst [22]). We noticed that a great enhancement in the MB decomposition rate by anatase TiO_2_ (0 mol% W), k_sunlight_/k_UV-A_ ≈ 1.7 (Figure 12b), can be explained by tge greater intensity of the UV-A component in sunlight as compared to the laboratory lamp. However, the W insertion further increased this ratio up to 6.5 for 15 mol% W, which can be a sign of photocatalyst activation by the visible light. Since WO_3_ does not crystallize in the material at W ≤ 15 mol%, this activation is believed to involve intraband impurity states. The activation of the W_x=0.15_-TiO_2_ nanocatalyst by sunlight can be an additional argument supporting its use in environmental catalysis.

## 4. Conclusions

Photocatalytic W-TiO_2_ nanopowders with a large variation in the composition x = C_W_/(C_W_ + C_Ti_), including end members TiO_2_ and WO_3_, were successfully synthesized by using the sol–gel method, using TTIP and WCl_6_ precursors and a low hydrolysis ratio h = C_H2O_/(C_Ti_ + C_W_) = 1.25, following calcination at 450, 500, 550 and 600 °C to form a crystalline product. The materials preserved the anatase crystalline structure, while the crystalline size decreased with an increase in W content up to 0.15 mol%; the nucleation of WO_3_ crystallites was observed with the greater W loadings. The structural characterizations suggest that the solubility limit of W into anatase TiO_2_ is 15 mol% W. The adsorption and photocatalytic kinetics of the prepared materials were evaluated based on MB removal in aqueous solutions. Both adsorption kinetics and adsorption capacity strongly increased in the material with 15 mol% W, in which the maximum adsorption capacity reached 0.25 mM/g, which is almost 50 times higher compared to that of pure anatase TiO_2_. Overall, the rates of MB adsorption and photocatalytic decomposition were stronger in, respectively, W-rich and Ti-rich compositions. A comparison of the photocatalytic activities under UV-A and sunlight illuminations revealed two optimal compositions with tungsten contents of W_1_ = 2 mol% and W_2_ = 15 mol%. Our analysis permitted us to attribute them to the adsorption/photocatalytic decomposition of MB molecules at the W-TiO_2_ photocatalyst surface. The normalization of decomposition rates for the adsorbed amount of MB showed (i) the conservation of the intrinsic TiO_2_ material’s activity at low doping 0 ≤ W ≤ 0.02 and (ii) a decrease in the activity with greater W insertion. The compensation for the activity decrease by the adsorption increase was observed in the material’s pollutant removal process.

The synergy of adsorption and photocatalysis in the water purification process was shown, which revealed the material with 15 mol% W to be the most effective in terms of pollutant removal. Furthermore, an additional activation of the W-TiO_2_ photocatalyst by sunlight compared to a UV-A lamp was shown. To summarize, the mixed oxide W_x=0.15_-TiO_2_ nanocatalyst appeared to be promising for applications in the environmental process of pollutant removal in aqueous media, especially in emergency cases involving a strong peak in the pollutant concentration.

## Figures and Tables

**Table 1:** Preparation method and photocatalytic activity of W-TiO_2_ nanomaterials.

Method	Optimal W Loading mol%	Illumination	Enhancement	Pollutant ^(1)^	Reference
Sol–gel	3	UV-A	2.1	Formic acid	[35]
Hydrothermal	1	Sunlight	~3	Rhodamine B	[52]
Sol–gel	2	Sunlight	2.1	Malathion pesticide	[38]
Physical mixing	10–15	UV-A/visible	2.0	Methylene blue and orange G	[50]
Sol–gel	1	UV-A	2.5	Formic acid	[36]
Sol–gel	0.02–1	UV-A	2.3	Cr_2_O_7_^2−^	[21]
Ultrasound- assisted	5.4	Visible	3.9	Methylene blue	[28]
PLD	- ^(2)^	Visible	-	Methylene blue	[43]

^(1)^ Aqueous solutions. ^(2)^ Multilayer WO_3_/TiO_2_ structure; optimum 5% WO_3_ in layers thickness.

## Data Availability

The raw/processed data required to reproduce these findings cannot be shared at this time as the data also form part of an ongoing study.

## Supplementary Materials

The following supporting information can be downloaded at: https://www.mdpi.com/article/10.3390/nano14090765/s1, Table S1: W/Ti elemental composition of selected W(x)-TiO_2_ nanopowders calcinated at 550 °C; Figure S1: W/(W + Ti) composition of W-TiO_2_ nanopowders (calcinated at 550 °C); Figure S2: BET plots of W-TiO_2_ nanopowders (calcinated at 550 °C); Figure S3: Semi-logarithmic plots of MB degradation kinetics using W-TiO_2_ photocatalyst (*C_catalyst_* = 0.125 g/L) under UV-A lamp (λ = 365 nm) (a–c) and sunlight (d) illuminations.
