# Supplementary material for: Mixed Metal Oxide W-TiO2 Nanopowder for Environmental Process: Synergy of Adsorption and Photocatalysis
Source: Nanomaterials (Basel). 2024 Apr 26;14(9):765. doi: 10.3390/nano14090765 (PMC11085299; doi:10.3390/nano14090765)
Supplement: Supplementary file 1 [file nanomaterials-14-00765-s001.zip › nanomaterials-2948565-supplementary.pdf]

nanomaterials

Supporting Materials

Mixed Metal Oxide W-TiO_2 Nanopowder for Environmental Process: Synergy of Adsorption and Photocatalysis

Khley Cheng [1], Socheata Heng [1], Siteng Tieng [1], Ford David [1], Sarah Dine [2], Oriana Haddad [2], Christophe Colbeau-Justin [3], Mamadou Traore [2] and Andrei Kanaev [2,*]

[1] Department of Chemistry, Royal University of Phnom Penh, Russian Blvd, Phnom Penh, Cambodia; chengkhley@yahoo.com (K.C.); socheataheng3579@gmail.com (S.H.); tiengsiteng@gmail.com (S.T.); forddavidaus@gmail.com (F.D.)
[2] Laboratoire des Sciences des Procédés et des Matériaux, CNRS, Université Sorbonne Paris Nord, 93430 Villetaneuse, France; sarah.dine@lspm.cnrs.fr (S.D.); oriana.haddad@cnrs.fr (O.H.); mamadou.traore@lspm.cnrs.fr (M.T.)
[3] Institut de Chimie Physique, CNRS UMR 8000, Université Paris-Saclay, 91405 Orsay, France; christophe.colbeau-justin@universite-paris-saclay.fr
* Correspondence: andrei.kanaev@lspm.cnrs.fr

1. EDX measurements

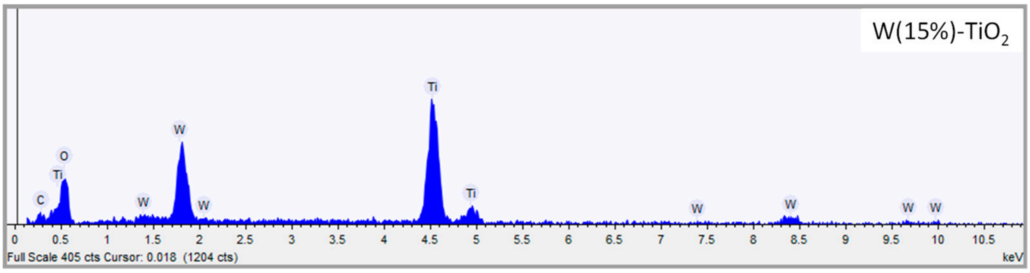

[(1)] Traces of C are due to supporting tape. No contamination was observed.

Table S1. W/Ti elemental composition of selected W(x)-TiO_2 nanopowders calcinated at 550 °C, where $x = C_W / (C_W + C_{Ti})$.

Samples	W(0.02)-TiO_2	W(0.04)-TiO_2	W(0.08)-TiO_2	W(0.15)-TiO_2	W(0.30)-TiO_2
**W / (W + Ti)**	0.0177	0.0376	0.0863	0.1403	0.3172

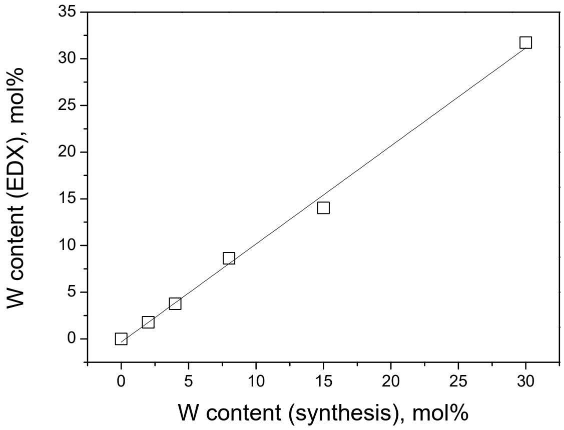

Figure S1. W/(W+Ti) composition of W-TiO_2 nanopowders (calcinated at 550 °C).

2. BET measurements

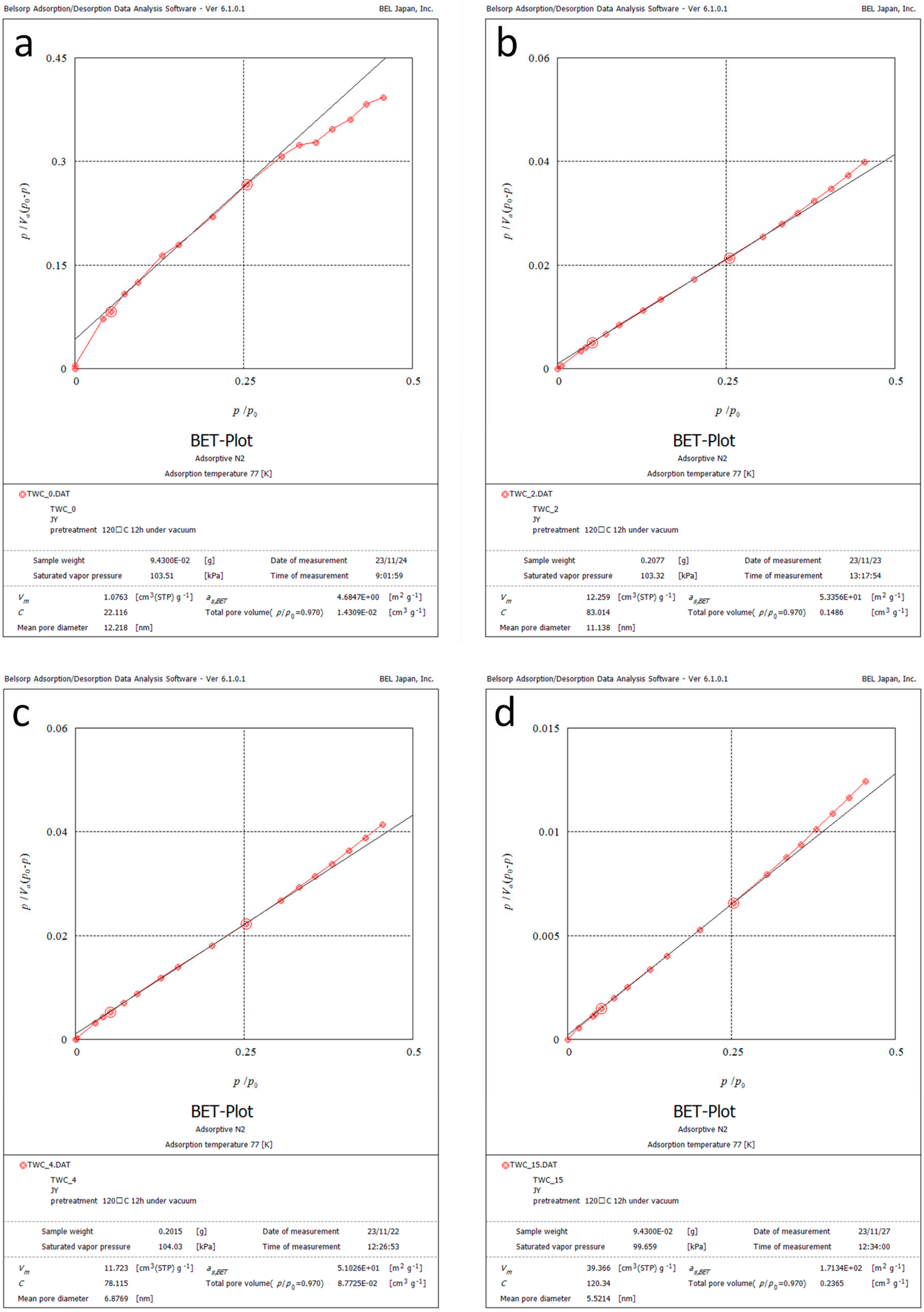

Figure S2. BET plots of W-TiO_2 nanopowders (calcinated at 550 °C) with W content of 0 (a), 2 (b), 4 (c) and 15 (d) mol%.

3. Kinetics of photocatalytic process

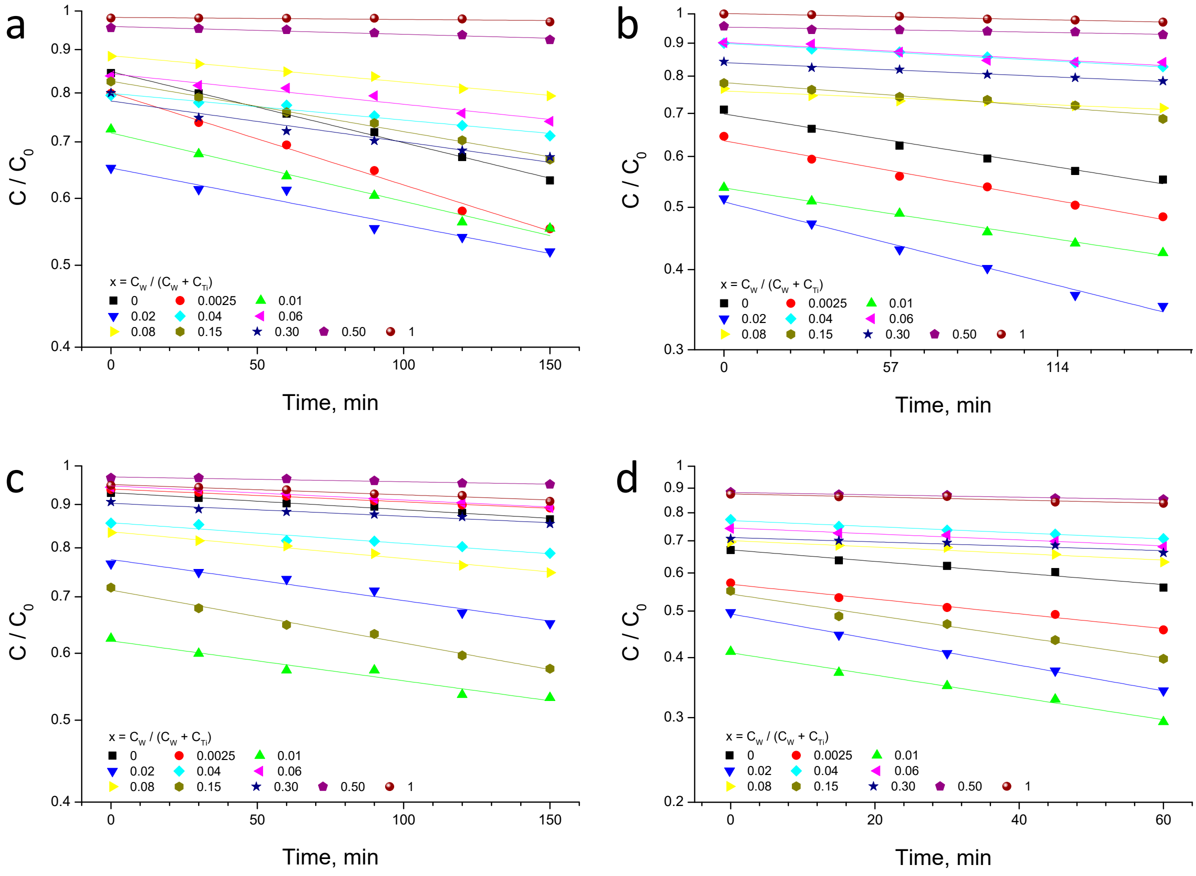

Figure S3. Semi-logarithmic plots of MB degradation kinetics using W-TiO_2 photocatalyst ($C_{catalyst}$=0.125 g/L) under UV-A lamp (λ=365 nm) (a-c) and sunlight (d) illuminations. Calcination temperatures are 500 °C (a), 550 °C (b, d), and 600 °C (c). Photocatalysts compositions x are labeled in inset. Fit of experimental data with first-order process kinetics is shown by solid lines.
